# Benchmark dataset for multi depot vehicle routing problem with road capacity and damage road consideration for humanitarian operation in critical supply delivery

**DOI:** 10.1016/j.dib.2022.107901

**Published:** 2022-02-02

**Authors:** Wadi Khalid Anuar, Lai Soon Lee, Stefan Pickl

**Affiliations:** a Department of Logistics and Transportation, School of Technology Management and Logistics, Universiti Utara Malaysia, Sintok, Kedah 06010, Malaysia; b Laboratory of Computational Statistics and Operations Research, Institute for Mathematical Research, Universiti Putra Malaysia, Serdang, Selangor 43400, Malaysia; c Department of Mathematics and Statistics, Faculty of Science, Universiti Putra Malaysia, Serdang, Selangor 43400, Malaysia; d Fakultät für Informatik, Universität der Bundeswehr München, Neubiberg 85577, Germany

**Keywords:** Multi depot routing problem, Road capacity, Humanitarian operations, Disaster, Chaotic event

## Abstract

The dataset for Multi Depot Dynamic Vehicle Routing Problem with Stochastic Road Capacity (MDDVRPSRC) is presented in this paper. The data consist of 10 independent designs of evolving road networks ranging from 14-49 nodes. Together with the road networks are the Damage file (DF) for each corresponding road network. The DF simulates the damage level of roads within the networks due to a disaster source, thus affecting travel time and road capacity. We applied this data to test our proposed algorithm and validate our proposed model. This dataset served as an addition to the Vehicle Routing Problem (VRP) datasets that specifically addressed the road capacity problem during a disaster from an epicentre and could be used for other applications that constitute chaotic events and compromised road networks.

## Specifications Table

**Table:** 

Subject	Applied Mathematics
Specific subject area	Vehicle Routing Problem in Operations Research
Type of data	Table
	Image
	Network Figure
How data were acquired	All test instances presented are simulated and the respective Damage Files (DFs) are generated based on these instances. This simulated dataset is inspired and derived from the 2015 Nepal Earthquake, reported by news reports, independent reports and scholarly articles, from which the information are gathered. Additionally, the geographical map of Nepal and the earthquake epicentre of the earthquakes are referred to when generating a concept instance. From the concept instance, other instances are developed with varying degrees of complexities to allow for sensitivity analysis. A related research [10] served as a general guide in developing the test instances. Other relevant information regarding humanitarian operations in regards to VRP is also referred ([1]).
Data format	Raw
Parameters for data collection	Parameters such as node placements and number of special nodes as well as road network are purposely varied in ways that would allow for sensitivity analysis. Some parameters are also derived from assumptions made for the model of the problem in deriving costs and time travels. ([2])
Description of data collection	A simulated road network on each instance is designed and developed based on observing the challenges reported during the event. The development of the networks is driven by the objectives in highlighting these challenges such that different scenarios could be simulated by ranging the instance from a simple instance to a high complexity instance in terms of computation effort. Assumptions were made when placing the nodes, the edges/ roads and determining the road capacities of the road. From the graph theory, the road networks are designed as an undirected, incomplete, connected graph to represent more realistic road networks. Furthermore, the earthquake tremor is assumed to be dispersed radially, with the radius chosen based on the Fibonacci sequence. The damage level of an edge is assumed to be correlated to the number of intersections observed between the radial tremor lines and the edge. The road capacity and damage value are determined such that the values can be served as initial values suitable for the dynamic and stochastic version of the problem applied. ([2])
Data source location	Dataset was designed and revamped from collective ideas in two institutions, local (Universiti Putra Malaysia) and abroad (University of Bundeswehr Munich) in an office environment with portable equipment.
Data accessibility	The data is accessible through Mendeley Data Repository
	Repository name: MDDVRPSRCV1_Test_Instance
	Data identification number: 10.17632/nt6j9c8653.1
	Direct URL to data: https://data.mendeley.com/datasets/nt6j9c8653/2
	Instructions for accessing these data: Dataset is accessible through the link given above. From there instructions are listed to download and configure the data.
Related research article	Some of the test instances in the proposed dataset are applied in the research paper:
	W. K. Anuar, L. S. Lee, H.-V. Seow, S. Pickl, A multi-depot vehicle routing problem with stochastic road capacity and reduced two-stage stochastic integer linear programming models for rollout algorithm, Mathematics 9 (2021). https://doi.org/10.3390/math9131572
	to validate the model and solution algorithm proposed.

## Value of the Data

• The presented data is the first problem instances that assign specifically road damages to each road in the road network based on the simulated earthquake tremor lines from an epicentre as well as road capacity in each problem instance. Through this data unlimited sets of simulated data can be generated due to random road capacity as observed in work [2]. Within minimal focus on road capacity and road damage for VRP researches, a general and standardized test data that address such problem is vital in order to compare and benchmark a proposed solution algorithm.
• Interested researchers who look into the VRP specifically on the road capacity and road damage problem in times of disaster when developing a model with multi depots and multi customers/shelters/connecting nodes in a chaotic environment can benefit from this data.
• The presented data is fully reusable and customisable for further insights and developments at every stage of the experiment through which different sets of simulated data can be generated:
  1. The test instances could be configured further for deeper insights by expanding selected networks into a more complex version of the original or reducing the networks to observe the basic operation of the intended model. Vehicle number of the heterogeneous or homogeneous fleet as well as their respective capacities could be manually varied to create different test instances based on the applied vehicle number.
  2. Unlike typical test instances, the damage level for each road/ edge is included in the data (DF), which is computed by the method described in [2]. This DF is generated in a python program, as explained in [2].
  3. The dataset is presented in ways that flexibility is allowed in terms of adjusting nodes coordinates and the capacity of the road for sensitivity analysis. Furthermore, one test instance is adequate as a reference for others to design their own road network. Moreover the test data can be used independently without the “Damage File” of earthquake tremor if so needed.
  4. The data is developed for the application of delivering medical supply during a post-earthquake disaster event. However, this data could also be used for other scenarios where road capacity and damaged road condition are concerned due to an event triggered at a single coordinate. For example, scenarios such as bomb evacuation, a mass outbreak during a pandemic or a huge concert that leads to congestions affecting road capacities could be simulated while computing for efficient vehicle routing.
  5. As opposed to test instances derived from real geographical locations and real road network, this dataset could be served as a basic dataset allowing for freedom in designing networks that highlight difficult aspects of a specific VRP to ensure a more robust solution algorithm and model development.

## 1 Data Description

This dataset, applied first for MDDVRPSRC ([2]), provides the following road network characteristics:

• Multiple depot nodes represent multi depots problems.
• Multiple shelter nodes represent demand locations with different demands.
• Connecting nodes represent junction points within the road networks.
• Edges represent roads within the road networks, each with it's respective capacity. The roads are divided into three: (1) Highways with the highest road capacity, (2) normal roads with a medium road capacity, (3) city roads with the least road capacity.
• An epicentre of an earthquake that spread the tremor outward radially affecting the road condition in terms of road capacity and travel time.

For a stochastic and dynamic road capacity as addressed by [2], this data provides the initial value of road capacities of the network and the damaged unit each road sustained due to the tremor of the earthquake.

This presented data applies basic parameters of MDDVRPSRC detailed in Table 1.

**Table 1:** Parameters and Variables for Presented Dataset adopted from ([1]).

Parameters	
N	connecting node set
D	depot set
S	shelter set
H	N⋃S⋃D
E	set of edges E={(i,j):i,j∈N⋃S⋃D,i≠j}
$w_{i}$	demand of emergency node or shelter i
M	set of vehicles
Q	maximum capacity of vehicles after replenishment at depot
$r_{i,j}$	deterministic road capacity $r_{i,j}\in Z^{★}$, where $Z^{★}=Z^{+}⋃\left\{0\right\}$
$p_{i,j}$	damage unit sustained by edge (i,j)
$C_{i,j}$	cost incurred if edge (i,j) is travelled
$T_{i,j}$	time travelled of edge (i,j)
G	road network in the form of Graph G

The presented data is accessible in the repository mentioned in the table (Data Specifications) above consist of 4 main files followed by a standard open software license (“LICENSE.txt”). These four files are listed below:

1. LOAD_INSTANCE NETWORK.zip
2. LOAD_DAMAGE NETWORK.zip
3. LOAD_INTS_DAMG_V5.py
4. README TEST_INSTANCE.txt

The LOAD_INSTANCE NETWORK.zip file can be extracted via open and commercial extraction tools where a folder denoted as “LOAD_INSTANCE NETWORK” is then extracted. Within this folder are the 10 test instances representing 10 different road networks. Each test instance in an excel file consists of 5 excel sheets within with names as listed below:

1. “parameter”
  • The numbers of the vehicles (|M|), depots (|D|), shelters (|S|) and connecting nodes (|N|) are specified.
  • User has the option to use the number of vehicle as specified in this sheet or manually change the number in the python file (LOAD_INTS_DAMG_V5.py line 188 – 192).
2. “dataC”
  • Here, the coordinate of node i is specified (given as $\left(i_{x},i_{y}\right)$) in the road network simulated in the Euclidean map.
  • Furthermore, the demand for each node $w_{i}$ is also specified.
3. “RoadCap”
  • In this Excel sheet, the deterministic road capacity $r_{i,j}$ is mapped where the node i and j are represented by the rows and columns, respectively, such that each row $i_{c}=i+1$ and column $j_{c}=j+1$ are in the matrix.
4. “DemandData”
  • lists the demand of each node in the increasing order of nodes.
5. “network”
  • attaches the road network as displayed in the python canvas generated in the work [2].

Moreover, each test instance are accompanied with respective (DF), which could also be found within a folder denoted as “LOAD_DAMAGE NETWORK” by extracting the second file in the repository(LOAD_DAMAGE NETWORK.zip). All DF excel file consist of 2 sheets:

1. “damages”
  • this excel sheet mapped all edges (i,j)∈E from the respective test instance with the associated damages $p_{i,j}$ sustained by the edges
2. “epicentre”
  • here the coordinate of the epicentre from which the simulated earthquake tremor lines are generated, thus causing damages to the roads/ edges as specified.

**Fig. 1:**
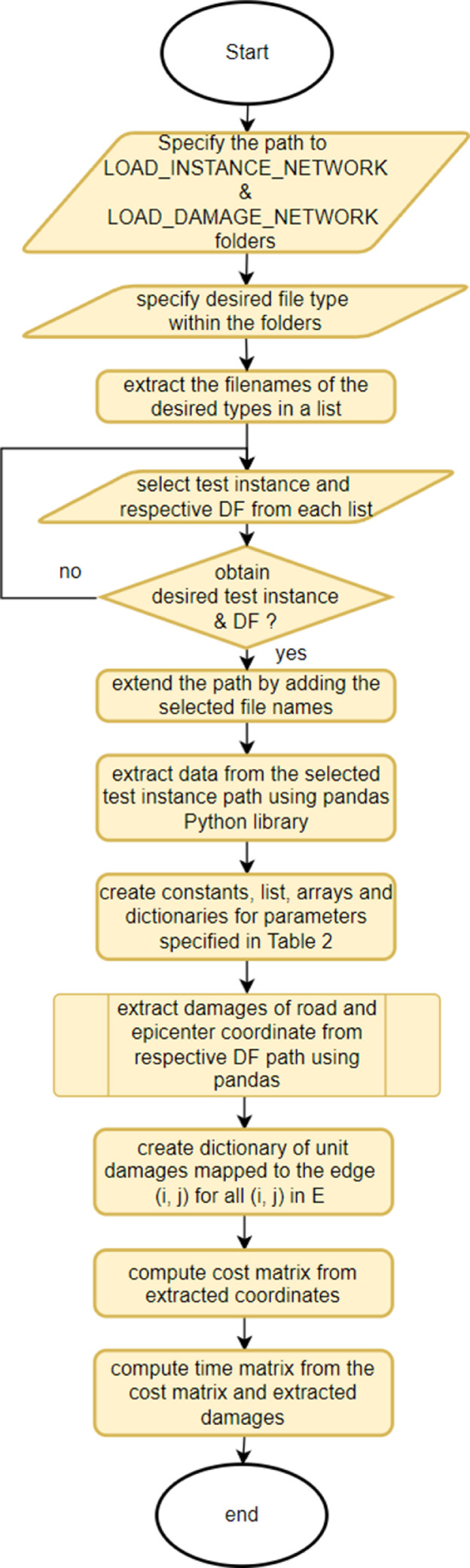
Flowchart of python file “LOAD_INTS_DAMG_V5.py” to extract data from selected test instance and respective DF.

Meanwhile, the data from the test instance and respective DF is uploaded when the python file “LOAD_INTS_DAMG_V5.py” is executed. The upload could be the initial part of developing VRP models in Python that addressed the problem associated with the multi depot, road capacity and road damages. The flowchart of “LOAD_INTS_DAMG_V5.py” in extracting and processing data from the selected test instance and respective DF is illustrated in Fig. 1.

Finally, the “README TEST_INSTANCE” is the first file to be downloaded and opened from the repository. This file consists of:

1. Appreciation note.
2. Introduction to the test instance and DF.
3. Authors and contributors and acknowledgement requests when utilizing this data.
4. brief overview of test instances.
5. brief overview of DF.
6. brief overview of “LOAD_INTS_DAMG_V5.py” Python file, the output that it produces including the cost and time matrix.
7. Requirements to process the data downloaded from the repository.
8. Instruction on downloading the file and adapting the “LOAD_INTS_DAMG_V5.py” Python file to the local user machine.

## 2 Experimental Design, Materials and Methods

### 2.1 Design

The proposed data is derived based on the information reported during 2015 Nepal earthquake event. Among these reports are the bottleneck problem ([8]), urgent medical supply demands ([11] and [9]), set-up of temporary shelters and field hospitals due to compromised buildings ([4] and [11]), damage to the road network ([13]) as well as the limited vehicle available for urgent medical supply delivery ([5]).

From these pieces of information, the proposed data in the form of test instances are derived from experimenting with specific approaches that could alleviate the problem. Such approaches include introducing:

• multi depots to address the bottleneck.
• split delivery to tackle the problem of the limited vehicle and routing when considering stochastic road capacity and delay time travel on compromised roads.
• setting up temporary emergency shelters near the event (epicentre) of disaster where affected victims are.
• emergency medical supply delivery routing while considering stochastic road capacity and damage effect on the roads.

The task of dataset development is divided into two:

1. Development of the road networks such that the solution approach from the proposed model and solution algorithm [2] could be tested.
2. Incorporating damage unit for each road within each road network due to simulated earthquake tremors to complete the dataset.

In the earlier phase of development, the road network of Nepal in [6] and the earthquake epicentres in [7] are referred to. From these sources, it is observed that the highways are mainly constructed near the country’s border, thus the outermost of the road network. The test instances are therefore designed by incorporating:

• City roads which are located in the innermost of the road network.
• Normal roads which are located roughly between the city roads and the highways.
• Multi depots which should be scattered at the outermost of the road network where highways are located.

Furthermore, based on [7], it is observed that the earthquake epicentres are located in the inner part of Nepal. The test instances are then further designed with the following specification:

• All emergency shelters should be located within the inner part of the road network.

### 2.2 Materials

The following materials were utilized to create the test instances:

• reporting materials ([8], [11] [9], [4], [13], [5], [6] and [7]) that provides the concept of the test instances.
• Microsoft Office Excel materializes the test instances in the form of simulated road networks.
• Python with essential libraries such as Tkinter ([12]) and Networkx ([3]) to draw the networks and verify the networks.

### 2.3 Method

Based on the specifications selected for the test instance design in Design subsection, the following steps are performed in producing the test instances:

1. A basic road network consists of 3 depot nodes, 3 emergency shelter nodes as well as 8 connecting nodes are first designed based on the Euclidean map in positioning the nodes. Their coordinates, along with their numbers and node assignments (depots, shelters, and connecting) are saved in a test instance file. In the test instance, a fixed vehicle number is given, although this is easily reconfigured when developing the model for manual number adjustment. The numbers of depots, shelters and connecting nodes, on the other hand, are fixed according to the specified test instance.
2. Edges is then drawn and listed in the same test instance file with the following considerations:
  (a) no direct connection among depots.
  (b) the road network is based on an undirected incomplete graph in Graph Theory such that the nodes are not fully connected amongst each other, and the edges are bidirectional.
  (c) no direct connection is allowed between depots and shelters such that a connecting node must at least be visited once.
3. Highway, normal road, and city road are next assigned for each edge with specifications mentioned in the Design subsection.
4. For each type of road, a deterministic road capacity is assigned, and the matrix of road capacity for possible pairs of nodes forming the edge between them is then added to the instance file.
5. Demand for each node is also assigned and added to the test instance. The demand is assigned such that:
  (a) for a minimum number of vehicles specified in the experiment (|M|=4), more than one trip is required.
  (b) the minimum demand of a shelter must be more than the fixed capacity of the vehicle (50 units in the experiment) to allow the experiment with the split delivery operation.
  (c) both depots and connecting nodes have zero demands.
6. The resulting test instance is then applied to the work [2] in Python:
  (a) data extraction from the test instance is inspected for potential errors. The test instance or the Python code is modified accordingly if any error is found.
  (b) cost matrix is automatically computed based on the Euclidean Distance formula based on the node’s coordinate given in the test instance.
  (c) time travel matrix is computed based on the assumption of constant speed of 90 km/hour.
  (d) the extracted data of the road network from the test instance is utilized using the Networkx Python library to recreate the road network in Python based on the Graph Theory:
    • the road network is an undirected incomplete connected graph G.
    • i and j represent nodes that form the edge (i,j)∈E.
    • and H is a set of all nodes in G.
    • Node s∈S⊂H is an emergency shelter node,
    • while node d∈D⊂H and n∈N⊂H represent depot node and connecting node respectively.
  (e) This network represented by the Graph G is then visualized using Python Tkinter library and compared with the network designed in Excel for any potential errors.
  (f) the applicability of the test instance for the work [2] is observed, and the required modification of the network is noted.
7. The process of improving the test instance and applying the improved version in step 6 is repeated until the functionality of the test instance is developed as desired. The resulting test instance is illustrated in Fig. 2.

**Fig. 2:**
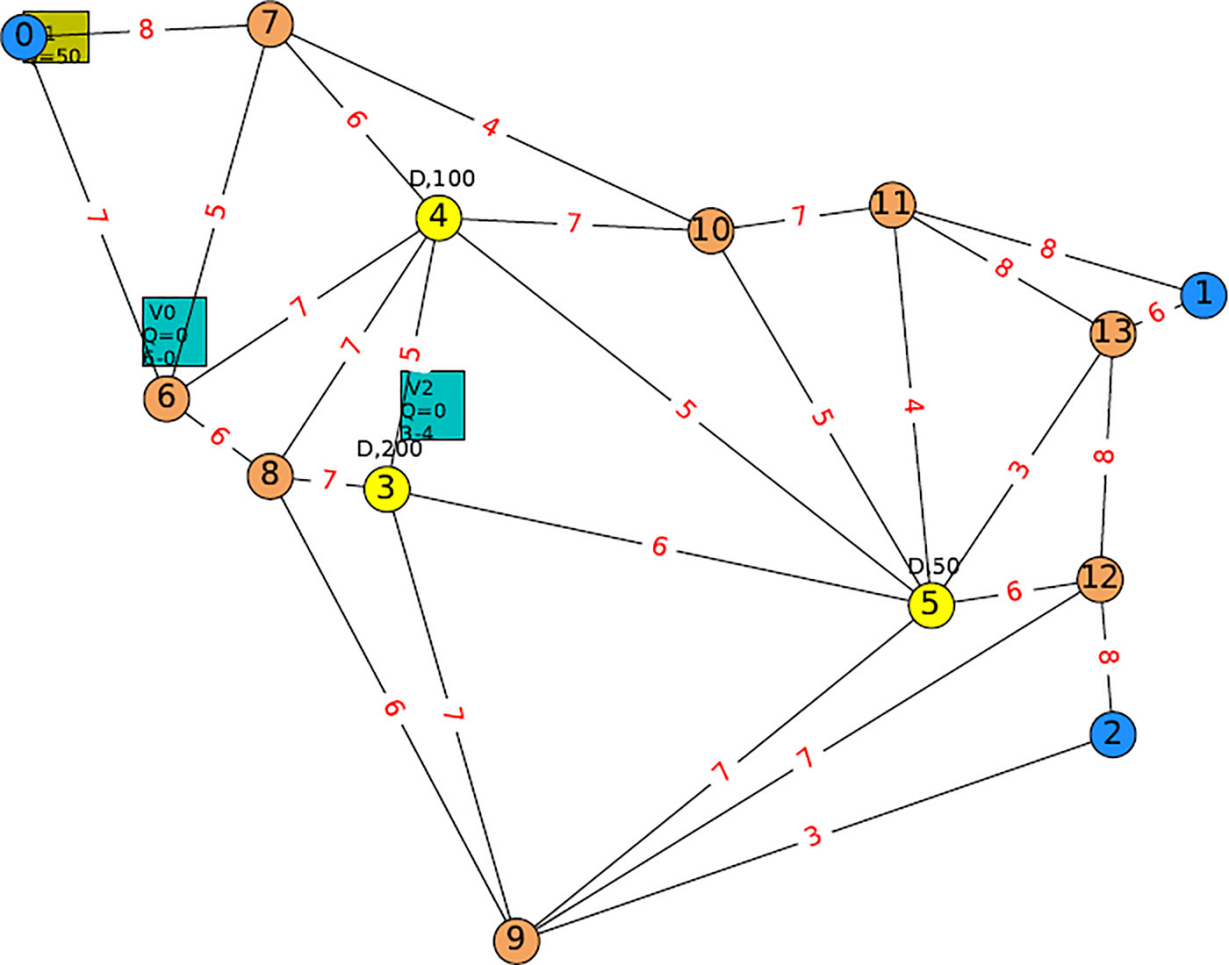
Visualization of Road Network based on Undirect Incomplete Graph.
8. Once the test instance is ready; the corresponding DF is then developed:
  (a) Assumption is made that the earthquake tremors damage some of the roads thus affecting the road capacity as well as the travel time along the road (for the case of deterministic, dynamic and stochastic road capacity problem).
  (b) The damage unit values are obtained through the simulated earthquake tremor lines described in Algorithm 1 in [2] The computed road capacities based on these values and the initial road capacity from the test instance in work [2] can be observed in Fig. 3 at the center of each respective edge.

**Fig. 3:**
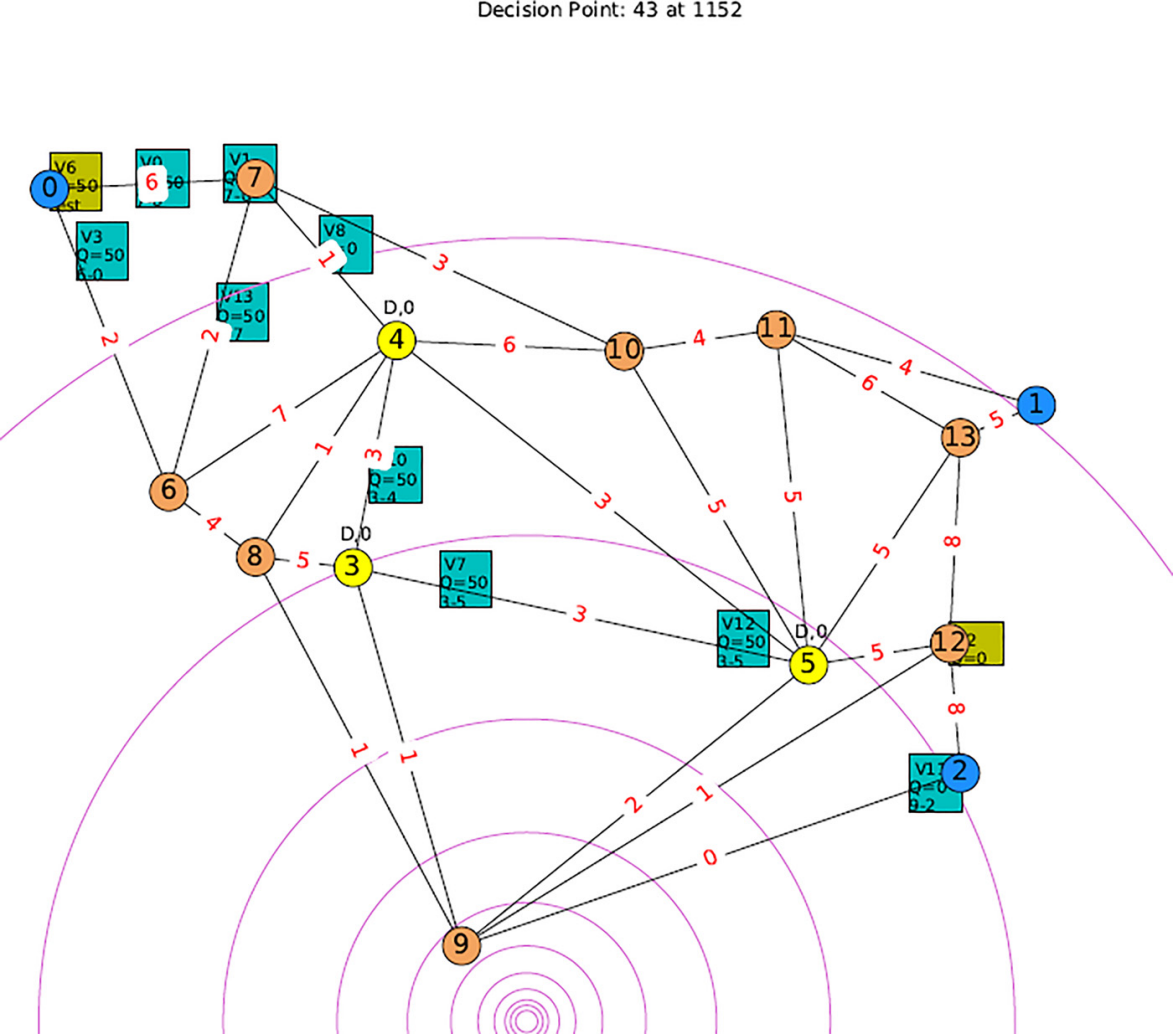
Simulating Earthquake Tremor and Computing Damage Unit for Each Road (The Value of Road Capacity Changes Dynamically for Dynamic and Stochastic Problem).
  (c) Similarly, the travel time along an edge should be longer not only due to the distance (length of the edge), but also due to the damage sustained by the edge. Therefore the time travel matrix computed in steps 6(c) is hence computed incorporating the damaged unit sustained by corresponding edges (Equation 13 in [2]).
  (d) Such proposed mechanism is advantageous when there are more than one epicentres within the road network to evaluate the condition of the roads.
9. Once the basic road network (DataD3N8S3) is validated, more increasingly complex road networks are developed by adding more edges and nodes to the basic road network. The 10 core test instances representing different road networks are listed in Table 2. In the table, the road capacity (6,7,8) represents the road capacity of city road, normal road, and highway, respectively. Each parameter listed in the table, including the road capacity tuple, could be changed to develop a new test instance.

**Table 2:** Test Instances.

Instance	Depot	Shelter	Nodes	Total Demand	Road Capacity
D3N8S3	3	3	8	550	6,7,8
D4N11S4	4	4	11	550	6,7,8
D4N30S10	4	10	30	1650	6,7,8
D5N13S5	5	5	13	650	6,7,8
D6N16S6	6	6	16	950	6,7,8
D7N18S7	7	7	18	1250	6,7,8
D8N20S8	8	8	20	1350	6,7,8
D8N22S9	8	9	22	1600	6,7,8
D9N25S10	9	10	25	1650	6,7,8
D9N30S10	9	10	30	1650	6,7,8
10. This test instances could be further expanded from the 10 core instances by differentiating the fixed vehicle numbers for the emergency delivery operation as is done in [2].

The test instances derived by following steps 1–10 could also be emulated if raw data is obtained. For example, a case study of an earthquake disaster in a known location with known numbers of delivery points (shelters), junctions (connecting nodes) and depots/warehouses along with their coordinates as well as comprehensive networks consisting of different types of roads could be adapted to the test instances instead of designing simulated networks. In this case, steps 1–5 could be directly applied by replacing hypothetical numbers with the raw data at hand. Step 6 can be applied to validate the real test instance with the exclusion of steps 6(b) and 6(c) if the raw data also includes cost and travel time data along each edge or road.

Additionally, if the raw data also includes damages measurement for each road in the network, then step 8(b) could be excluded when designing the test instance based on the raw data. Furthermore, the epicentre coordinate is not needed as it is only required to simulate the earthquake tremor lines.

Once the test instance and the corresponding DF is produced, more hypothetical complex test instances could be developed by altering the raw data. The resulting test data would then have the advantage of being based on raw data from existing topography. Furthermore projected plans such as building future depots could be applied on top of these raw data to simulate practical hypothetical scenarios.

Despite the benefit of incorporating raw data when developing test instances and DFs, the methodology provided in this section allows for more freedom in designing any networks required for experimentations which could potentially be very useful for education and planning. The theoretical mathematical model of VRP such as MDDVRPSRC and the solution approach to the problem could be validated to any degree of setup for further insights and developments.

## Ethics Statement

This work meets the requirements of ethics as stated in (https://www.elsevier.com/journals/data-in-brief/2352-3409/guide-for-authors) and (https://www.elsevier.com/about/policies/publishing-ethics#Authors). This work also does not involve studies with animals and humans.

## CRediT authorship contribution statement

**Wadi Khalid Anuar:** Conceptualization, Methodology, Software, Validation, Formal analysis, Investigation, Resources, Data curation, Writing – original draft, Writing – review & editing, Visualization. **Lai Soon Lee:** Conceptualization, Methodology, Software, Validation, Formal analysis, Investigation, Data curation, Writing – original draft, Writing – review & editing, Supervision. **Stefan Pickl:** Conceptualization, Methodology, Supervision.

## Declaration of Competing Interest

The authors declare that they have no known competing financial interests or personal relationships which have, or could be perceived to have, influenced the work reported in this article.
